# Stable potassium isotope ratios in human blood serum towards biomarker development in Alzheimer's disease

**DOI:** 10.1093/mtomcs/mfae038

**Published:** 2024-08-31

**Authors:** Brandon Mahan, Yan Hu, Esther Lahoud, Mark Nestmeyer, Alex McCoy-West, Grace Manestar, Christopher Fowler, Ashley I Bush, Frédéric Moynier

**Affiliations:** Melbourne Analytical Geochemistry, School of Geography, Earth and Atmospheric Sciences, University of Melbourne, Melbourne, Australia; IsoTropics Geochemistry Lab, Earth and Environmental Science, James Cook University, Townsville, Queensland 4814, Australia; Institut de Physique du Globe de Paris, Université Paris Cité, CNRS, 75238 Paris, France; Department of Geoscience, University of Nevada, Las Vegas, Las Vegas, NV 89154, USA; Institut de Physique du Globe de Paris, Université Paris Cité, CNRS, 75238 Paris, France; IsoTropics Geochemistry Lab, Earth and Environmental Science, James Cook University, Townsville, Queensland 4814, Australia; IsoTropics Geochemistry Lab, Earth and Environmental Science, James Cook University, Townsville, Queensland 4814, Australia; Melbourne Analytical Geochemistry, School of Geography, Earth and Atmospheric Sciences, University of Melbourne, Melbourne, Australia; The Florey Institute of Neuroscience and Mental Health, University of Melbourne, Melbourne, Australia; The Florey Institute of Neuroscience and Mental Health, University of Melbourne, Melbourne, Australia; Institut de Physique du Globe de Paris, Université Paris Cité, CNRS, 75238 Paris, France

**Keywords:** potassium, isotope, Alzheimer's disease, metallomics, biomarker

## Abstract

The Alzheimer's disease (AD)-affected brain purges K with concurrently increasing serum K, suggesting brain–blood K transferal. Here, natural stable K isotope ratios—δ^41^K—of human serum samples were characterized in an AD biomarker pilot study (plus two paired Li-heparin and potassium ethylenediaminetetraacetic acid [K-EDTA] plasma samples). AD serum was found to have a significantly lower mean δ^41^K relative to controls. To mechanistically explore this change, novel *ab initio* calculations (density functional theory) of relative K isotope compositions between hydrated K^+^ and organically bound K were performed, identifying hydrated K^+^ as isotopically light (lower δ^41^K) compared to organically bound K. Taken together with literature, serum δ^41^K and density functional theory results are consistent with efflux of hydrated K^+^ from the brain to the bloodstream, manifesting a measurable decrease in serum δ^41^K. These data introduce serum δ^41^K for further investigation as a minimally invasive AD biomarker, with cost, scalability, and stability advantages over current techniques.

## Graphical Abstract

**Graphical Abstract:**
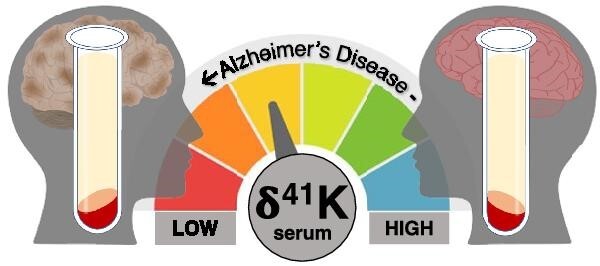
Human blood serum stable K isotope compositions (δ^41^K) indicate statistically significant light K isotope enrichment in Alzheimer's disease (AD) relative to controls. This marker displays good performance in identifying AD (receiver operating characteristic curve analysis), introducing serum δ^41^K for further exploration as a minimally invasive AD biomarker.

## Introduction

Changes in brain biometals in Alzheimer's disease (AD) have led to their increasing exploration in biological systems.^1–3^ This has drawn attention from the field of *isotope metallomics*, which utilizes analytical (geo)chemistry techniques to characterize the abundance and distribution of biometal isotopes in biological systems.^4–11^ For biometals in relation to neurodegenerative diseases such as AD, it has been observed that metals including Ca, Fe, Cu, and Zn accumulate in the brain as a function of age and/or the development of neurodegenerative disorders (e.g. AD), where most metals are linked to the presence and/or aggregation of amyloid β (Aβ) fibrils and the development of senile plaques.^1,12–26^ Complementary research has indicated that the AD-affected brain also expresses deficits of certain metals, namely here, K,^3^ and that this may occur prodromally (e.g. midlife).^27^

Brain metal accumulation with neurodegeneration has led to the investigation of changes in total metal levels in the bloodstream as potential diagnostic metrics for AD, on the premise that metal dyshomeostasis in the brain might manifest a correlative change in blood fractions, thereby possibly serving as non-invasive biomarkers and diagnostic indicators. However, on the blood side of the blood–brain barrier, elemental abundances are more subject to exogenous and endogenous confounders such as sample processing/storage, environmental exposures, homeostatic transport mechanisms, genetics, and cultural differences, and therefore the use of blood metal abundances alone as indicators of disease can be variable and at times contradictory; see Acevedo *et al*.^26^ and Harris and Fahrenholz,^28^ and especially Babic Leko *et al.*^29^ and references therein. Furthermore, sample stability during storage and transport is a recognized and further emerging issue for many organic biomarkers, e.g. polypeptide-based approaches,^30–34^ beckoning for diagnostic tools that are less sensitive to these constraints, such as with inorganic mass spectrometry, where stability of blood fractions is of no consideration because samples are fully digested, atomized, and ionized prior to analysis.

The natural abundance of metal isotopes in biological systems can be altered by: (i) changes in their bonding environments during exchange reactions (equilibrium isotope fractionation, e.g. healthy vs diseased cells), where stronger bonds favour heavier isotopes^8,35^; and/or (ii) [non-equilibrium] kinetic effects during dominantly unidirectional processes such as diffusion.^36,37^ In both cases, isotope fractionation can be well-described and modelled through *ab initio* theoretical calculations, namely density functional theory^38–40^ for equilibrium isotope fractionation, allowing for mechanistic interpretations of empirical and experimental data.^10,41,42^ In brief, for equilibrium isotope fractionation, the isotopic composition of a given bodily reservoir, especially in relation to others (e.g. blood relative to brain tissue), is beholden to bonding environment, whereas for kinetic isotope fractionation, lighter isotopes tend to become enriched in reaction products along a chain of [unidirectional] chemical reactions.^39,40^ In AD, observations indicate that metals such as Ca, Fe, Cu, and Zn accumulate in the brain due to changed bonding environment (binding e.g. to Aβ), and these metals are hypothesized to play a mechanistic role in AD pathology; therefore, most elemental and isotopic studies to date have focused on these metals,^17,43,44^ with Cu and Zn showing promise for utility in AD diagnostics.^8,35,45^ Where available, results from *ab initio* calculations of equilibrium isotope fractionation agree with the direction and general magnitude of isotope fractionation in this context, even considering that such calculations simplify the bonding environment to that of amino acids as approximations for more complex protein binding sites.^46^

For K, previous research has reported a significant decrease in the brain with AD, with a correlative increase in serum K,^3^ indicating a linkage between the two and the possibility for developing a serum AD biomarker based on K and its isotopes. In Roberts *et al.*,^3^ total K concentrations in human AD brain homogenates decreased by >20% (24.4% decrease from ∼2 mg/g wet weight in the control group), with an average concomitant increase in serum K of 2.6% (from ∼145 mg/L). A key diagnostic pathology in AD is the formation of amyloid plaques by extracellular deposition of Aβ, but the soluble Aβ pool (i.e. that not yet aggregated into insoluble fibrils) is also indicative of disease severity.^47^ Increased K intake has been linked to reduced risk of dementia (especially vascular) in humans,^48^ and to reduced oxidative stress in an amyloid precursor protein/presenilin-1 (APP/PS1) murine model for AD (with decreased Aβ aggregation and reduced *tau* phosphorylation).^49^ Related work on the association between Aβ and K in humans reported a linkage between low K intake at midlife and low Aβ_42_ in cerebrospinal fluid in late life, further suggesting a biological and/or pathological link between K and AD (and Aβ), notably in the prodromal phase of the disease.^27^ The possibility that K systematics earlier in life are related to AD risk later in life is also supported by separate research linking increased serum K to mild cognitive impairment,^50^ corroborated by the independent observation that decreased K in the AD brain correlates with increased K in blood serum.^3^ Lastly, our recent work^51^ found a linkage between K brain concentrations and K isotopic compositions—^41^K/^39^K relative to a standard, denoted as δ^41^K in per mil (per thousand), ‰—in porcine AD models at midlife, wherein it was hypothesized that efflux of hydrated K^+^ from the brain with AD (due to the presence of Aβ) would manifest as a light K isotope excursion (lower δ^41^K) in the bloodstream. Taken together, these findings point to a connection between perturbed K metabolism and AD, likely present in the prodromal phase, indicating that serum K and its isotopes might serve as minimally invasive biomarker tools for understanding and diagnosing AD. It is noted that exogenous influences—diet in particular—on individual bodily reservoir isotopic compositions are yet not well constrained and will require future investigation (see Sullivan *et al.*^52^ and references therein).

The analytical challenges inherent to K stable isotope ratios measurements have only been operationally overcome in recent years.^53–56^ Innovative analytical methods such as new-generation collision–reaction cell, multi-collector inductively coupled plasma mass spectrometers (CRC-MC-ICP-MS) have opened new research avenues in the study of natural variations of K stable isotope ratios.^54,55,57,58^ Particularly relevant here, Moynier *et al.*^54^ showed that accurate and high-precision δ^41^K (<0.03‰ uncertainty) can be achieved with only 125 ng (or less) of K, opening up the possibility to isotopically characterize minute amounts of even low K concentration samples. To date, the only published data for K isotopes in blood fractions are in Moynier et *al.*,^55^ Cui *et al*.,^58^ Hobin et *al.*,^59^ Tacail *et al.*,^60^ Hobin *et al*.,^61^ and Higgins *et al*.^62^ While K isotope data are limited, K concentrations in most bodily reservoirs (e.g. plasma, organs, and brain) are two or more orders of magnitude higher than that of transition metals (e.g. Fe, Cu, and Zn) (e.g. Albarede *et al.*^63^ for human serum^9^; for porcine organs and blood fractions), marking K as an attractive tracer in both practical and analytical terms. That is, K isotope compositions in these reservoirs are more accessible due to much higher typical concentrations (thus also less susceptible to contamination), and far less sample is needed to generate statistically robust isotopic measurements, especially by CRC-MC-ICP-MS where much less analyte K is necessary for reliable measurements compared to conventional methods not using the collision–reaction cell.^54,55,58^

In the context of blood biomarker development, because typical K concentrations in the brain are generally over an order of magnitude higher than that in blood plasma/serum (e.g. ≈3,000 ppm compared to 100–200 ppm, respectively),^9,63^ the isotopic signal of K disruption in the brain (especially that which may purge K into extracellular space) plausibly could be detected in the bloodstream, supported by the size of the previously observed excursion in absolute brain K concentrations in AD,^3^ and previous observations that organ-biofluid differences in δ^41^K can be quite large under healthy conditions,^58^ meaning that a change in one reservoir can impart a measurable difference in another (especially moving from higher to lower concentration), the base logic in the application of K isotopes to human disease.

In the present study, we test the potential of human serum K isotope compositions for AD biomarker development (as hypothesized in Mahan *et al.*^51^) by characterizing serum K isotope compositions.

## Methods

### Sample collection and digestion

In total, 20 serum samples were analysed, from 10 unique AD subjects and 10 unique control (CN) subjects; Li-heparin and potassium ethylenediaminetetraacetic acid (K-EDTA) anticoagulated plasma samples from two AD cases were analysed to compare their paired serum values to determine whether plasma values differ to serum due to addition of anticoagulants. Cryogenically frozen blood serum and plasma samples (∼500 μl) were obtained from the Australian Biomarker & Lifestyle Flagship Study of Ageing (AIBL) through The Florey Institute of Neuroscience and Mental Health, University of Melbourne, Australia), with ethics committee approval both by St Vincent's Health (HREC 028/06) and the James Cook University (JCU) Human Ethics Committees (HREC H8650). The AIBL cohort is 95% Northern European Caucasian (with remainder largely Southern European). Controls are unrelated to AD and were randomly selected from a CN pool who have remained longitudinally cognitively normal. All AD subjects had been clinically diagnosed via the Mini-Mental State Exam (MMSE) assessment and specialist panel review using National Institute of Neurological and Communicative Disorders and Stroke and the Alzheimer’s Disease and Related Disorders Association (NINCDS-ADRDA) criteria, with biomarker confirmation through positron emission tomography (PET) centiloid scores (Table 1); CN subjects were cognitively unaffected and had subdiagnostic brain amyloid on PET scan (null criteria fitting).^64^ Despite prior work indicating that age has no major effect on K isotope compositions in mammals,^60^ all subjects were age-matched to within 15 years and with identical average ages for both AD and CN (75 years old) (Table 1). Due to limited sample availability, it was not possible to match sex ratios for the samples; however, previous work has indicated that sex does not significantly affect K isotope systematics in mammals.^60^ There were serum samples from seven males and three females in the AD group, and from two males and eight females in the CN group.

**Table 1.** ID, clinical status, demographic, Mini-Mental State Exam (MMSE) scores, PET centiloid values, and δ^41^K for human serum samples in this pilot study

**AIBL ID**	**Diagnosis**	**Sex**	**Age (year)**	**MMSE**	**PET centiloid**	**δ^41^K (‰)**	**2σ**	***n***
1994	AD	Male	83	17	166.4	−0.50	0.05	4
1994dup						−0.50	0.04	4
1984	AD	Male	72	21	122.2	−0.82	0.08	3
2079	AD	Male	77	24	97.7	−0.26	0.05	4
2064	AD	Male	76	28	107.8	−0.15	0.04	5
2084	AD	Female	72	22	47.1	−0.55	0.04	3
2086	AD	Male	84	21	116.5	−0.68	0.04	5
2086dup						−0.71	0.06	4
2526	AD	Female	70	27	110.6	−0.38	0.07	4
2447	AD	Female	68	23	126.4	−0.45	0.04	4
2486	AD	Male	72	25	142.1	−0.97	0.06	3
2279	AD	Male	74	23	79.3	−0.67	0.08	3
Average			75	23	112	−0.55		
Median			73	23	114	−0.53		
2SD			11	6	66	0.46		
2049	CN	Female	77	29	−1.2	−0.36	0.09	4
2049dup						−0.33	0.08	4
2021	CN	Male	87	27	−3.7	−0.32	0.02	4
2021dup						−0.31	0.06	4
2056	CN	Female	76	30	−3.7	−0.31	0.02	3
2093	CN	Female	72	28	6.9	−0.37	0.07	3
2058	CN	Female	71	28	−7.1	−0.18	0.03	3
2066	CN	Female	75	29	−0.2	(−0.77)	0.05	3
1912	CN	Male	76	29	6.1	−0.45	0.03	5
1868	CN	Female	71	29	−7.3	−0.30	0.06	4
1868dup						−0.28	0.05	4
1869	CN	Female	72	28	−2.3	−0.47	0.03	3
2090	CN	Female	68	29	1.9	−0.18	0.06	5
Average			75	29	−1	−0.32		
Median			74	29	−2	−0.31		
2SD			10	2	10	0.18		
FBS1						−1.60	0.02	3
FBS2						−1.63	0.06	4
FBS3						−1.63	0.03	3
Average						−1.62		
2SD						0.04		

Notes: () denotes statistical outlier, and ‘*n*’ denotes analytical replicates within the same analytical session.

The digestion protocol has been adapted and optimized from previous work.^9,51,54,58,65^ All samples were digested in an approximate 1:10 mixture of concentrated hydrogen peroxide (30% H_2_O_2_) and concentrated double-distilled nitric acid (70% HNO_3_) in 30 ml of ultra-clean polyfluoralkyl (PFA) vials, in three sequential and cumulative steps. First, serum samples were added to PFA vials without the use of pipettes (to avoid contamination), typically equating to ∼400 μl of serum; to this 100 μl of H_2_O_2_ and 2.0 ml of HNO_3_ were added and left loosely capped and unheated for 30 min (‘soft oxidation’), followed by heating at 120°C on a hotplate for 24 h tightly capped. A further 250 μl of H_2_O_2_ and 1.0 ml of HNO_3_ were added, samples tightly capped, and heated at 120°C for 24 h. A final 50 μl of H_2_O_2_ and 100μl of HNO_3_ were added, samples tightly capped, and heated at 120°C for 24 h. This equates to final volumes of 400 μl of H_2_O_2_ and 3.1 ml of HNO_3_, for a total digest volume of ∼3.5 ml (digestion is not fully conservative due to outgassing). After determination of average K concentrations by ICP-MS using a 500-μl aliquot of the final digests (see the ‘Results’ section and *Supplementary Data*), further aliquots of 400 μl were taken from the final digest (∼11%, equating to 35–40 μl of serum) and pipetted into ultra-clean 7-ml PFA vials for dispatch to Institut de Physique du Globe de Paris (IPGP) for further processing. Half of each aliquot (∼20 μl) was used for K chemistry and isotopic analyses using post-Cu extraction solutions, adopting a similar robust sample conservation approach as that taken for precious low-quantity cosmochemical samples (see Hu *et al.*^56^ and references therein). Assuming even low-end K concentrations in serum of ∼100 ppm K, ∼20-μl aliquots equate to ∼2 μg or more of K, i.e. enough for several repeat analyses in 100–150-ng/g analyte solutions (and four times this in 25-ng/g solutions). Aliquots were dried down at 80°C on a hotplate inside a sealed evaporation chamber and sent to IPGP for K separation chemistry and isotopic analysis by CRC-MC-ICP-MS (Nu Sapphire™).

All statistical analyses were conducted using GraphPad™ Prism™ (Mac v9.50) through licence at the University of Melbourne.

### Potassium separation chemistry and isotopic analysis

Potassium separation chemistry and isotopic analyses follow previously established robust methods.^54,58^ At IPGP, dried sample aliquots were redissolved in 0.5-mole/litre (M) HNO_3_ in preparation for potassium isolation through cation exchange chromatography. In brief, BioRad® Poly-Prep™ columns were loaded with 2 ml of pre-cleaned Bio-Rad AG® 50W-X8 resin (200–400 mesh) and conditioned with 10 ml of 0.5 M HNO_3_. Sample solutions were then loaded on the resin in 1 ml of 0.5 M HNO_3_. Matrix elements are eluted with 13 ml of 0.5 M HNO_3_, with K isolates subsequently collected in another 22 ml of 0.5 M HNO_3_. In between column passes (3×), the resin was stripped of any remaining sample ions by eluting 10 ml of 6 M HCl through the column.

Potassium stable isotope measurements were carried out at IPGP using the Nu Sapphire™ CRC-MC-ICP-MS, where the collision cell pathway was used with H_2_ gas to neutralize Ar^+^ and ArH^+^ species, removing these as major mass interferences for K isotope measurements.^54,55,66^ Given the large amount of K present in our solutions (>2 μg), standard and sample solutions were introduced to the instrument as 100–150-ng/g (ppb) solutions with an ESI® Apex™ Omega desolvating system fitted with an integrated 100-μl/min PFA nebulizer/probe assembly (ESI® MicroFlow™ nebulizer), and standard Ni dry cones at the instrument interface. In line with convention, K isotope compositions (^41^K/^39^K, denoted as δ^41^K in per *mil* notation, or per thousand, ‰) were measured using standard–sample bracketing, with National Institute of Standards and Technology Standard Reference Material (NIST SRM)-3141a used as the natural abundance bracketing standard for direct comparison to other works.^54,55^ The K stable isotope compositions expressed in ‰ as δ^41^K are formulated as follows:


\begin{eqnarray*}
{\mathrm{\delta }}{}_{}^{41}{\mathrm{K}} = \left( {\frac{{{}_{}^{41}{\mathrm{K}}/{}_{}^{39}{{{\mathrm{K}}}_{{\mathrm{sample}}}}}}{{{}_{}^{41}{\mathrm{K}}/{}_{}^{39}{{{\mathrm{K}}}_{{\mathrm{SRM}}3141{\mathrm{a}}}}}} - 1} \right){\mathrm{\ }} \times {\mathrm{\ }}1000,
\end{eqnarray*}

where ^41^K/^39^K refers to the measured abundance ratios. In this relativistic framework, sample-to-sample comparisons (both being defined relative to a standard composition) are discussed in terms of being isotopically lighter (or heavier) than one another (where lighter denotes relative enrichment in ^39^K, the lighter K isotope, and therefore lower δ^41^K). In general, sample analyte solutions were analysed four to six times (minimum three) to generate within-session reproducibility metrics. All uncertainties herein have been conventionally reported as two times the standard deviation (2σ).

### *Ab initio* calculation of K isotope fractionation between relevant bonding environments

For further evidence-based interpretation of the current work, and to test hypotheses drawn out of previous work,^51^ we conducted a subset of *ab initio* calculations to predict K isotope fractionation between its hydrated form (with and without solvation effects) and when molecularly bound to aspartate and glutamate, such as is the case for K in Na/K-ATPase (the activity of which is altered in AD).^67^ Additional calculations were included for K-EDTA as a common additive in prepared biological samples, as well as for other biologically relevant forms of bound K.

Vibrational frequencies of metal complexes were calculated after successful geometry optimization in ORCA 5.0.3.^68^ Calculations were performed with density functional theory using the PBE0 functional^69^ and the def2-svp all-electron basis set^70^ for all elements. The numerical integration grid ‘defgrid3’ was used, convergence tolerance for self-consistent field was to set 1.0E^9^ Eh, and for geometry optimization to 2.0E^−7^ Eh. Calculations of amino acid metal complexes were performed *in vacuo* with K bound to carboxyl groups. Most recent experimental measurements show a hydration number of 6 for K^+^ in aqueous solution.^71,72^ Therefore, we modelled aqueous K^+^ with six water molecules in its first hydration shell. For vibrational frequency calculation, K was substituted by the isotopes ^39^K and ^41^K using the masses 38.9637069 and 40.96182597, respectively.^73^ Reduced partition function ratios (β-factors) were then calculated using the equation^74^:


\begin{eqnarray*}
\beta = {{\left( {\mathop \prod \limits_i^{3N - 6} \frac{{{{u}_i}}}{{u{{^{\prime}}_i}}}\ \frac{{\frac{{{\mathrm{exp}} \left( { - \frac{{{{u}_i}}}{2}} \right)}}{{1 - {\mathrm{exp}}\left( { - {{u}_i}} \right)}}}}{{\frac{{\exp \left( {\frac{{ - {{{u^{\prime}}}_i}}}{2}} \right)}}{{1 - {\mathrm{exp}}\left( { - {{{u^{\prime}}}_i}} \right)}}}}} \right)}^{\frac{1}{n}}}
\end{eqnarray*}

with


\begin{eqnarray*}
{{u}_i} = \frac{{hc{{\omega }_i}}}{{kT}},
\end{eqnarray*}

where *h* is Planck's constant, *c* is speed of light, *k* is Boltzmann constant, *ω_i_* is *i*^th^ of 3*n* − 6 vibrational frequencies, *T* is absolute temperature, *n* represents the number of atoms in the species, *N* represents the total number of atoms, and *u′* refers to the lighter isotope. In complexes with multiple K atoms, all K atoms were substituted by the identical isotope and β was subsequently normalized by *n* number of K isotopes.^75^ Cartesian coordinates of optimized K-bearing species for *ab initio* calculations can be found in *Supplementary Data* (Tables S2–S9).

## Results

**Fig. 1:**
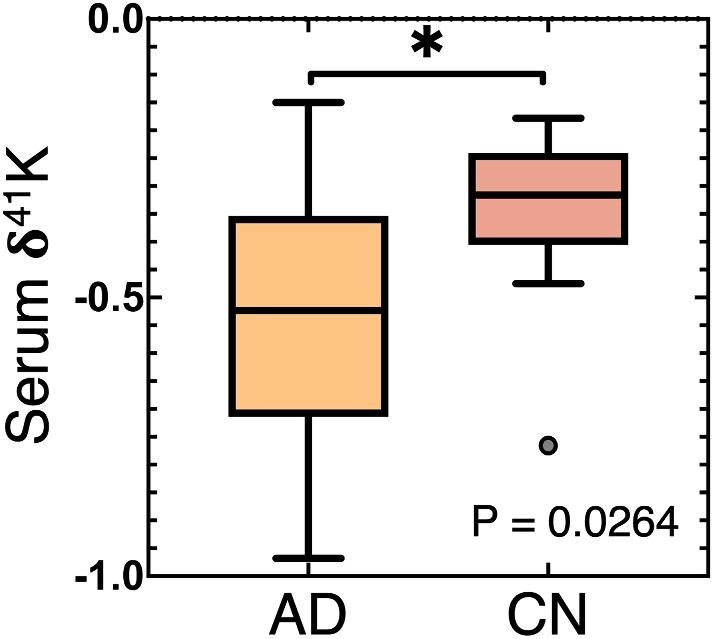
Boxplot of ^41^K/^39^K isotope ratios—δ^41^K in per mil, ‰—for Alzheimer's disease (AD) versus control (CN) subjects, with Welch's *t*-test P result reported. Outlier(s) indicated by grey circles.

Potassium isotope compositions, as δ^41^K relative to NIST-3141a, are reported in Tables 1 and 2 (serum and plasma data, respectively; uncertainties conventionally reported as two times the standard deviation, 2σ), along with neuropsychological testing (MMSE) and PET centiloid results and diagnosis. In total, 10 AD and 10 CN subjects were interrogated (Table 1 and Fig. 1). For reference, K concentrations determined by ICP-MS are reported in *Supplementary Data* alongside MMSE, PET centiloid, and δ^41^K (Table S1). As there are currently no certified reference materials (CRMs) with K isotope compositions (as certified values; however, see Moynier *et al.*^54^ for K isotope characterization of biological CRMs utilizing the same analytical approach herein), several full procedural replicates of a commercially available foetal bovine serum (FBS, Sigma Aldrich) were processed alongside sample batches as a matrix-matched control and to ensure no K isotope fractionation from ion exchange chromatography; all three FBS replicates returned δ^41^K values that are the same within analytical uncertainty (Table 1). Analytical duplicates (same K isolate, separate analytical session; five in total) were run across the analytical sessions to ensure there was no between-session analytical artefacts; all analytical duplicates returned δ^41^K values that are the same within analytical uncertainty (Table 1). Lastly, the overall mean for control samples of −0.32 ± 0.18‰ (2σ of all CN subjects, excluding one outlier, see below) is in excellent agreement with the previously determined value of −0.30 ± 0.04‰ for the pooled human blood serum CRM Seronorm™ Trace Elements Serum L-1,^59^ further validating the chemical and analytical techniques employed (see the ‘Discussion’ section). Typical analytical resolution within the current study is ±0.05‰.

**Table 2.** ID, clinical status, neuropsychological evaluation scores, PET centiloid values, and δ^41^K for paired serum–plasma samples; suffix ‘L’ denotes treatment with Li-heparin, and suffix ‘E’ denotes treatment with K-EDTA

**AIBL ID**	**Diagnosis**	**Sex**	**Age (year)**	**MMSE**	**PET centiloid**	**δ^41^K**	**2σ**	***n***
2526	AD	Female	70	27	110.6	−0.38	0.07	4
2526-L						0.01	0.08	3
2526-E						−0.03	0.03	4
2486	AD	Male	72	25	142.1	−0.97	0.06	3
2486-L						−0.61	0.02	3
2486-E						−0.13	0.04	3

The initial dataset was screened for outlier rejection and effects of anticoagulants prior to further statistical analyses. Paired plasma samples treated with Li-heparin and K-EDTA returned markedly different δ^41^K values than their serum counterparts and trended towards heavier values (Table 2) and are thus excised from statistical analyses. An outlier, AIBL 2066 (CN) was identified and excised from statistical analyses, leaving 19 data (10 AD and 9 CN). AIBL 2066 (CN, δ^41^K = −0.77 ± 0.05‰) was first identified as a statistical outlier by falling outside the 2σ (95%) envelope for CN δ^41^K values, with statistical outlier status confirmed by both the Tukey method (1.5 × interquartile range [IQR]) and the Grubbs test (α = 0.05); it has been maintained elsewhere in the current work for transparency and further discourse (see the ‘Discussion’ section). Both AD and CN pools were assessed for a possible sex effect, with neither returning a statistically significant effect (P = 0.7489 and 0.3933, respectively). No statistically significant correlation was found between δ^41^K and MMSE or PET centiloid, with Pearson's *R*^2^ values of 0.21 and 0.18, respectively; for comparison, MMSE versus PET Pearson's *R*^2^ = 0.62.

Overall, potassium isotope compositions, δ^41^K, ranged from −0.97 to −0.15‰ (min/max both defined by AD results), for reference, a large range comparable in magnitude to that seen across Earth's major geological reservoirs.^76^ As illustrated in Fig. 1, mean AD serum is isotopically lighter for K (average δ^41^K = −0.55 ± 0.46‰, 2σ) relative to controls (δ^41^K = −0.32 ± 0.18‰, 2σ), with a mean difference of 0.23‰; it is noted that the offset towards lighter K isotope compositions in AD versus CN subjects is not significantly affected and is still >0.2‰ if median values are chosen as representative. To determine a nominally healthy range for δ^41^K, 1.5 × IQR was re-calculated after outlier removal, yielding a value of 0.10‰ and therefore a healthy δ^41^K range of −0.22 to −0.42‰ for δ^41^K. To confirm that AD and CN data herein do not deviate significantly from Gaussian populations, a D'Agostino & Pearson normality test was applied, yielding P = 0.9692 and 0.9361 for AD and CN (respectively). To interrogate the comparative variance between AD and CN, an *F*-test was applied, yielding an *F* value of 6.0 and P = 0.019 (*), indicating unequal variance. Due to unequal variance between AD and CN, a Welch's *t*-test approach was applied to compare means, yielding P = 0.0264 (*). Additionally, the sensitivity and specificity of δ^41^K as a method of detecting AD were interrogated using receiver operating characteristic (ROC) curve analysis, yielding an area under curve (AUC) of 0.8 (good/very good).^77^ Applying the lower threshold value of −0.42‰ for ‘healthy’ δ^41^K to ROC results yields a sensitivity of 70% and a specificity of 89% in identifying AD. In summary, blood serum from AD subjects has a significantly lighter potassium isotope composition (lower δ^41^K) than that of CN; additionally, CN data cluster tightly around −0.3‰, while AD data display greater dispersion. While not a focus of the current work, serum K concentrations were collected for most samples to constrain aliquoting approach (6 AD and 10 CN); AD serum K was slightly higher than that in CN (1.9%); however, this was not statistically significant (P = 0.6166, equal variance unpaired *t*-test).

**Fig. 2:**
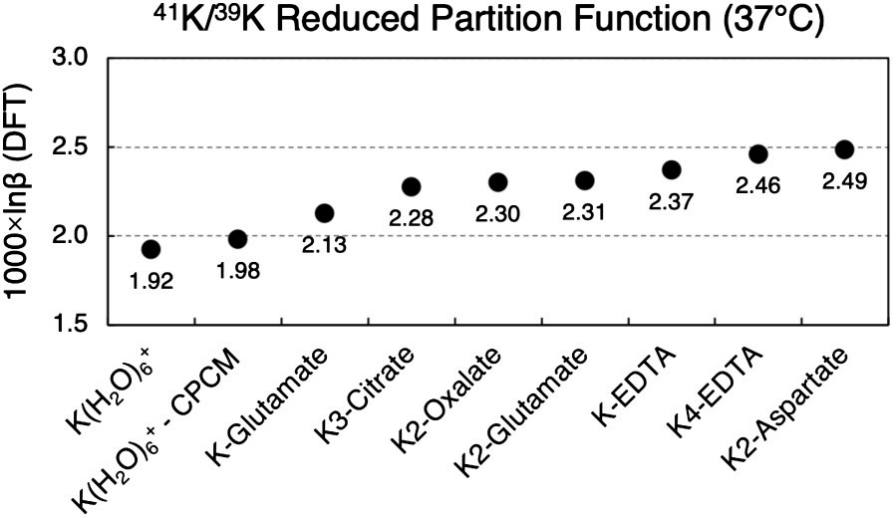
^41^K/^39^K reduced partition function for various biologically relevant species at 37°C. Relative isotopic enrichment can be predicted by subtraction; e.g. at normal body temperature, six-coordinated hydrated K^+^ is predicted to be 0.39‰ lighter than K_2_-glutamate (1.92–2.31 = −0.39).

Results of *ab initio* calculations to predict isotope fractionation of K between its hydrated form (six-coordinated hydrated K^+^; the conductor-like polarizable continuum model (CPCM) version includes solvation effects) and relevant molecularly bound environments are reported in Fig. 2. Calculation results are reported as 1000× the natural logarithm of the reduced partition function, or 1000 × lnβ, for δ^41^K (^41^K/^39^K); lower values indicate light isotope enrichment relative to higher values (i.e. lower δ^41^K refers to relative enrichment in ^39^K, and *vice versa*). In this reference frame, relative isotope compositions can be calculated by subtraction (e.g. 1.92–2.13 = −0.21‰; six-coordination hydrated K^+^ is 0.21 ‰ lighter than K-glutamate; Fig. 2). In line with the underlying quantum mechanical energy considerations governing equilibrium stable isotope fractionation, as well as previous *ab initio* calculations for other metals,^7,46,78^ theoretical predictions dictate that hydrated K^+^ is isotopically lighter than that more strongly bound in organic compounds. Hydrated K^+^ was the most isotopically light species of all *ab initio* calculations undertaken herein, meanwhile glutamate and aspartate (as in Na/K-ATPase) impel some of the heaviest K isotope compositions (0.2 and 0.4‰ higher, respectively).

## Discussion

Acknowledging that this is a pilot study (20 unique δ^41^K values, 10 AD and 10 CN), the significant changes in serum δ^41^K that we observed in AD may encourage future biomarker development.

Summarizing from above, blood serum from AD subjects has a statistically significant lighter potassium isotope composition (lower δ^41^K) than that of CN, with ROC curve analysis indicating good performance of this tool in predicting AD within the cohort.

Previous work in porcine brain tissue samples has indicated heavy K isotope enrichment in the brain associated with Aβ accumulation, and with relative brain K depletion compared to brains with low Aβ accumulation.^51^ The magnitude of K isotopic change in these samples displayed further qualitative correlation with temporal onset of brain changes between brain regions, e.g. the brainstem displayed larger δ^41^K excursion and is affected earlier in AD progression, whereas the amygdala displayed no change in δ^41^K and is affected later (and non-bilaterally) in AD (Zanchi *et al.*^79^ and Ji *et al.*,^80^ respectively). Cumulatively, the observations of Mahan et al.^51^ led to the hypotheses therein that:

- Aβ-induced efflux of hydrated ‘free’ K^+^ from the brain (see Roberts *et al.*^3^ and Yu *et al.*^81^) would be isotopically light relative to molecularly bound K, similar to that predicted by *ab initio* calculations for other metals (e.g. Moynier *et al.*^7^ for Zn isotopes within the context of AD); and
- Because K is much more abundant in the brain than in blood, the isotopic signature of purged hydrated K^+^ from the brain due to AD may transfer a measurable signal to the bloodstream as a light K isotope excursion,^51^ in the serum fraction.^82,83^

*Ab initio* results (1000 × lnβ values; Fig. 2) confirm that hydrated K^+^ is isotopically light relative to K-glutamate and K-aspartate (by 0.2 to 0.4‰, respectively; only slightly less if including solvation effects). These results corroborate the hypotheses put forth by Mahan et al.,^51^ and agree with the direction and magnitude of difference in δ^41^K for AD serum in the current work, being on average ∼0.2‰ lighter than CN (Table 1 and Figs. 1 and 2). The linkage between K dysregulation in the precursor stage of AD (e.g. at midlife)^27^ indicates that a change in serum δ^41^K might be detectable prior to clinical or pathological changes.

δ^41^K of paired Li-heparin and K-EDTA-treated samples trended towards heavier values. While it was not possible to mechanistically constrain observations for Li-heparin-treated plasma, *ab initio* results for K-EDTA yielded some of the highest ^41^K enrichment (second only to K_2_-aspartate), likely explaining the heavier δ^41^K of plasma samples treated with K-EDTA relative to paired serum (Table 2). Additionally, the average CN human serum δ^41^K of −0.32 ± 0.18‰ agrees very well with the −0.30 ± 0.04‰ determined for Seronorm™ Trace Elements Serum L-1.^59^ While further baseline work is needed, this may indicate that −0.3‰ is nominally representative of serum δ^41^K for healthy individuals (at least for Northern European Caucasian demographics). Lastly, while the increase of ∼2% in AD serum K concentrations determined herein is in line with previous observations by Roberts *et al*.^3^ (2.6%), data interpretation is cautionary as there was no statistical significance herein (P = 0.6166), whereas the dataset of Roberts *et al*. ^3^ contained over 1000 serum data with concentrations being the sole focus of their analytical methodology.

While the above provides a consistent and useful interpretative framework, it is noted that two AD δ^41^K values are heavier than the average control value of −0.32‰. This indicates that hydrated efflux of K^+^ from the brain may not fully explain the data and there are other unknown endogenous or exogenous inputs influencing K isotope compositions in AD, and this should be a focus of future work. The work of Hobin *et al.*^61^ observed a possible sex effect (endogenous) on serum K isotope compositions in 10 healthy mice (5 male and 5 female)—where there was a trend towards lighter δ^41^K in healthy female mice—and hypothesized intra-/extracellular K balance disparities and/or the estrous cycle as possible causes. No statistical sex effect was observed within the current work. This lends to at least two possibilities for future work to resolve: (i) this effect is not present in humans; or (ii) the light δ^41^K excursion in AD observed herein is a minimum, given that the AD pool has a slight male bias. If human and murine reservoirs are directly comparable, this future work will also allow for further data integration to understand general K isotope distribution across mammalian bodily reservoirs, e.g. the collation of data herein with that of Cui et al.^58^

If dietary effects persist through the stochastic homogenization of larger datasets, future cumulative baseline work can account for dietary effects through relative normalization, e.g. normalizing serum δ^41^K values to another accessible bodily reservoir (e.g. erythrocytes, tissue, and/or urine), as previously suggested in Mahan et *al.*^9^ (for Zn isotopes), and recently applied to δ^41^K in Tacail et al.^60^ and Higgins *et al*.^62^

Finally, the ROC curve with an AUC of 0.8 and a sensitivity/specificity of 70%/89% (respectively) indicates promising performance of δ^41^K in identifying AD. As benchmark comparisons to proteomics-based diagnostics, modern plasma Aβ_42_/Aβ_40_ ratios yield sensitivities in the range of ∼65–90% and specificities of 80–95%,^84,85^ with plasma P-tau181 sensitivities ranging from 80% to 95% and specificities from 75% to 80% depending on the Braak stage (e.g. Janelidze *et al.*^86^; AUC 0.85–0.90) (ranges herein are indicative but non-exhaustive). In this context, ROC performance metrics of δ^41^K in identifying AD are within the range of these proteomics-based approach techniques.

## Conclusions and outlook

A total of 20 human serum samples were characterized for their K isotope composition, δ^41^K in per mil (‰), from 20 subjects (10 AD and 10 CN) within the AIBL study (plus two paired Li-heparin and K-EDTA plasma samples). Anticoagulants Li-heparin and K-EDTA were observed to markedly alter δ^41^K compared to the paired serum value, and therefore it is concluded that data from such samples cannot be pooled with data from serum here or in the future.

The tight clustering of CN serum δ^41^K around −0.32‰, and its close agreement with data for pooled human serum determined elsewhere (−0.30‰), indicates that δ^41^K ≃ −0.3‰ is a current best estimate for nominally healthy human serum (at least for Northern European Caucasians). Statistical analyses returned a resolvable difference between AD and CN subjects, with the former having lighter (lower) δ^41^K values (*, P = 0.0264, Welch's *t*-test). An ROC curve with an AUC of 0.8, and a sensitivity of 70% and a specificity of 89%, indicates promising performance of δ^41^K in identifying AD. This is within reported performance metric ranges of modern proteomics-based diagnostics, and moreover the method described herein is based on inorganic mass spectrometry, and therefore is not susceptible to sample stability issues during transport and storage, e.g. organic breakdown/alteration. Together with novel *ab initio* predictions of K isotope fractionation, we hypothesize that the observed difference is due to enhanced hydrated K^+^ in AD serum, possibly reflecting a failure in brain Na/K-ATPase. This may reflect efflux of hydrated K^+^ from the brain because of Aβ, and given the early (midlife) link between K and Aβ, stable K isotopes might serve as an early biomarker for AD, and one which is robust with respect to sample stability, as well as being cost-effective and with great potential for translation and scalability.

These findings prompt the investigation of a much larger cohort of subjects for serum δ^41^K to enhance statistical resolving power and further interrogate any possible endogenous (e.g. age, sex, and genetics) or exogenous (e.g. diet and sampling) influences. Larger and more diverse subject cohorts in future work will also allow for combination of δ^41^K with other blood-based indicators, with plasma biomarkers—Aβ_42_/Aβ_40_, p-tau181, and p-tau217—being particularly useful (e.g. Fandos *et al.*,^84^ Doecke *et al.*,^85^ and Brickman *et al*^87^) given the known linkage between prodromal K dysregulation and Aβ.^27^

## Supplementary Material

mfae038_Supplemental_File

## Data Availability

The data underlying this article are available in the article and in its online supplementary material.
