# Supplementary material for: Stable potassium isotope ratios in human blood serum towards biomarker development in Alzheimer's disease
Source: Metallomics. 2024 Aug 31;16(9):mfae038. doi: 10.1093/mtomcs/mfae038 (PMC11411773; doi:10.1093/mtomcs/mfae038)
Supplement: mfae038_Supplemental_File [file mfae038_supplemental_file.docx]

**Supplementary Data to**

**Stable potassium isotope ratios in human blood serum towards biomarker development in Alzheimer’s disease**

Brandon Mahan, Yan Hu, Esther Lahoud-Heilbronner, Mark Nestmeyer, Alex McCoy-West, Grace Manestar, Christopher Fowler, Ashley I. Bush and Frédéric Moynier

This file contains:

**Supplementary Material-1**. ICP-MS methodology and *Table S1*: K concentrations, MMSE, PET Centiloid, and δ^41^K data for AD and CN

**Supplementary Material-2**. *Tables S2-S9*: Cartesian coordinates of optimized K-bearing species

**Supplementary Material-1**

**ICP-MS Methodology for [K] determination**

Analysis of K concentrations was conducted using triple quadrupole inductively coupled plasma mass spectrometry (TQ-ICP-MS; Thermo Fisher iCAP TQ) in the Advanced Analytical Centre at JCU (AAC; JCU Townsville). Sample aliquots were diluted to a final dilution factor of approximately 60 (gravimetrically monitored). Potassium concentrations were measured using Ar as the carrier gas and Ru as the internal standard. Ruthenium was chosen as the internal standard for matrix effects and mass bias (drift) correction due to its median ionisation thereby providing a reliable correction for matrix driven changes in signal. A five-point calibration protocol was developed using High Purity Standards™ HPS Trace Element 68 A and B solutions to provide robust analytics (blank as zero point, 10 ppb, 100 ppb, 500 ppb, 1ppm); as quadrupole sensitivity is linear over several orders of magnitude, this was deemed sufficient for K concentrations determined herein, with typical RSDs below 3% for all analyses. Calibration solutions were also analysed as unknowns during the sample sequences to determine and correct for instrument drift during the sequence. Multiple internal rinse cycles were incorporated to account for the possibility of remnant organics in the matrix.

**Table S1** - K concentrations, MMSE, PET Centiloid, and δ^41^K data for AD and CN

| **AIBL ID** | **Diagnosis** | **K (ppm)** | **MMSE** | **PET Centiloid** | **𝛅^41^K (‰)** |  |
| --- | --- | --- | --- | --- | --- | --- |
| 1994 | AD | 222 | 17 | 166.4 | -0.50 |  |
| 1994dup |  |  |  |  | -0.50 |  |
| 1984 | AD |  | 21 | 122.2 | -0.82 |  |
| 2079 | AD |  | 24 | 97.7 | -0.26 |  |
| 2064 | AD |  | 28 | 107.8 | -0.15 |  |
| 2084 | AD |  | 22 | 47.1 | -0.55 |  |
| 2086 | AD | 195 | 21 | 116.5 | -0.68 |  |
| 2086dup |  |  |  |  | -0.71 |  |
| 2526 | AD | 207 | 27 | 110.6 | -0.38 |  |
| 2447 | AD | 203 | 23 | 126.4 | -0.45 |  |
| 2486 | AD | 256 | 25 | 142.1 | -0.97 |  |
| 2279 | AD | 197 | 23 | 79.3 | -0.67 |  |
| ***Average*** |  | ***213*** | ***23*** | ***112*** | ***-0.55*** |  |
| ***Median*** |  | ***205*** | ***23*** | ***114*** | ***-0.53*** |  |
| ***2SD*** |  | *46* | **6** | **66** | ***0.46*** |  |
|  |  |  |  |  |  |  |
|  |  |  |  |  |  |  |
| 2049 | CN | 197 | 29 | -1.2 | -0.36 |  |
| 2049dup |  |  |  |  | -0.33 |  |
| 2021 | CN | 222 | 27 | -3.7 | -0.32 |  |
| 2021dup |  |  |  |  | -0.31 |  |
| 2056 | CN | 211 | 30 | -3.7 | -0.31 |  |
| 2093 | CN | 205 | 28 | 6.9 | -0.37 |  |
| 2058 | CN | 207 | 28 | -7.1 | -0.18 |  |
| 2066 | CN | 198 | 29 | -0.2 | -0.77 |  |
| 1912 | CN | 236 | 29 | 6.1 | -0.45 |  |
| 1868 | CN | 193 | 29 | -7.3 | -0.30 |  |
| 1868dup |  |  |  |  | -0.28 |  |
| 1869 | CN | 196 | 28 | -2.3 | -0.47 |  |
| 2090 | CN | 221 | 29 | 1.9 | -0.18 |  |
| **Average** |  | **209** | ***29*** | ***-1*** | **-0.32** |  |
| ***Median*** |  | 206 | ***29*** | ***-2*** | **-0.31** |  |
| ***2SD*** |  | 28 | **2** | **10** | **0.18** |  |
|  |  |  |  |  |  |  |

**Supplementary Material-2**

**Tables S2 to S9** - Cartesian coordinates of optimised K-bearing species

**Table S2** K_2_-Aspartate

| Element | X | Y | Z |
| --- | --- | --- | --- |
| O | -0.024035 | -1.584026 | 0.989710 |
| O | -1.369848 | -0.791759 | -1.506409 |
| O | 1.918726 | -0.949910 | 0.098129 |
| O | -2.566988 | 0.523850 | -0.165490 |
| N | 1.540378 | 1.677107 | 0.931924 |
| C | 0.418312 | 0.778084 | 0.740043 |
| C | -0.317779 | 1.153460 | -0.568500 |
| C | 0.820353 | -0.707008 | 0.646873 |
| C | -1.526413 | 0.251973 | -0.798818 |
| H | -0.297005 | 0.905159 | 1.569201 |
| H | 0.396833 | 1.082012 | -1.405673 |
| H | -0.638025 | 2.200546 | -0.475922 |
| H | 2.286346 | 1.330925 | 0.324091 |
| H | 1.905619 | 1.546544 | 1.874512 |
| K | -2.544831 | -1.782468 | 0.809213 |
| K | 0.676658 | -2.277308 | -1.665073 |

**Table S3** K_3_-Citrate

| Element | X | Y | Z |
| --- | --- | --- | --- |
| O | -0.835997 | -0.291457 | 1.457221 |
| O | 0.888089 | -2.264820 | 1.134563 |
| O | 0.507075 | -2.416102 | -1.055645 |
| O | 2.476471 | 0.258236 | 0.545472 |
| O | -2.577143 | 1.538384 | -1.236562 |
| O | 1.104274 | 1.895368 | 1.195845 |
| O | -3.008561 | 0.751223 | 0.803391 |
| C | -0.542899 | -0.618682 | 0.113397 |
| C | 0.373004 | 0.467244 | -0.557206 |
| C | -1.867251 | -0.696671 | -0.676869 |
| C | 0.308023 | -1.916262 | 0.068407 |
| C | 1.421035 | 0.944573 | 0.436710 |
| C | -2.597734 | 0.621691 | -0.399875 |
| H | -0.248768 | 1.292919 | -0.931490 |
| H | 0.825423 | 0.003730 | -1.444483 |
| H | -1.655733 | -0.836219 | -1.743027 |
| H | -2.455644 | -1.548465 | -0.303135 |
| H | -1.796438 | 0.013466 | 1.453896 |
| K | 3.000045 | -2.097704 | -0.461099 |
| K | 1.277546 | -0.394697 | 2.905081 |
| K | -1.386004 | 2.857314 | 0.830137 |

**Table S4** K-EDTA

| Element | X | Y | Z |
| --- | --- | --- | --- |
| O | 4.209281 | -2.432834 | -1.381135 |
| O | 1.006521 | 1.278678 | 2.640459 |
| O | -3.954438 | -1.814505 | 1.689078 |
| O | -0.332662 | 2.251523 | -1.124348 |
| O | 2.608481 | -1.042840 | -2.080651 |
| O | 0.746063 | 2.765086 | 1.012470 |
| O | -2.601044 | -0.067847 | 1.952302 |
| O | -2.223429 | 2.496604 | 0.052164 |
| N | 1.798187 | -0.591760 | 0.658470 |
| N | -1.641987 | -0.452560 | -0.771585 |
| C | 0.426130 | -1.013506 | 0.541244 |
| C | -0.262128 | -0.889175 | -0.843448 |
| C | 2.835049 | -1.493170 | 0.299197 |
| C | 2.071374 | 0.818136 | 0.538780 |
| C | -2.611368 | -1.290607 | -0.171222 |
| C | -2.060801 | 0.702198 | -1.535259 |
| C | 3.172108 | -1.606588 | -1.179893 |
| C | 1.217533 | 1.675247 | 1.489809 |
| C | -3.022430 | -0.962737 | 1.259253 |
| C | -1.560481 | 1.947780 | -0.816782 |
| H | 0.366455 | -2.055712 | 0.902494 |
| H | -0.162366 | -0.418456 | 1.256698 |
| H | 0.291070 | -0.154118 | -1.440655 |
| H | -0.184596 | -1.847590 | -1.397688 |
| H | 2.596659 | -2.514480 | 0.648761 |
| H | 3.777661 | -1.228146 | 0.813861 |
| H | 1.968997 | 1.210909 | -0.490200 |
| H | 3.120019 | 0.993369 | 0.838356 |
| H | -2.267067 | -2.342414 | -0.152583 |
| H | -3.556714 | -1.319737 | -0.747724 |
| H | -3.158491 | 0.734868 | -1.576771 |
| H | -1.665452 | 0.673173 | -2.567212 |
| H | 4.367538 | -2.451102 | -2.337673 |
| H | -4.178275 | -1.565661 | 2.598998 |
| H | 0.175878 | 2.653972 | -0.282178 |
| K | -1.424925 | 2.312501 | 2.525245 |

**Table S5** K_4_-EDTA

| Element | X | Y | Z |
| --- | --- | --- | --- |
| O | 4.708022 | -0.991600 | -1.805568 |
| O | 0.783254 | 1.027916 | 2.842035 |
| O | -4.738578 | -1.052477 | 1.825975 |
| O | -0.792339 | 1.062743 | -2.735229 |
| O | 3.506527 | 0.880036 | -1.756737 |
| O | 1.392566 | 2.409036 | 1.194111 |
| O | -3.546816 | 0.826362 | 1.824238 |
| O | -1.421362 | 2.404531 | -1.062186 |
| N | 1.710106 | -0.378212 | 0.117162 |
| N | -1.712308 | -0.397691 | -0.029767 |
| C | 0.586948 | -1.217197 | 0.540377 |
| C | -0.578319 | -1.220731 | -0.455483 |
| C | 2.610357 | -1.126042 | -0.730160 |
| C | 2.389594 | 0.248012 | 1.250238 |
| C | -2.610856 | -1.162912 | 0.804229 |
| C | -2.390913 | 0.233273 | -1.160716 |
| C | 3.691893 | -0.340820 | -1.472500 |
| C | 1.468412 | 1.329955 | 1.844549 |
| C | -3.717396 | -0.393472 | 1.526123 |
| C | -1.481932 | 1.336783 | -1.732919 |
| H | 0.941235 | -2.253967 | 0.726313 |
| H | 0.230724 | -0.839596 | 1.510432 |
| H | -0.224726 | -0.832642 | -1.422412 |
| H | -0.920239 | -2.260316 | -0.648304 |
| H | 2.021370 | -1.620346 | -1.523803 |
| H | 3.128742 | -1.944164 | -0.184878 |
| H | 3.305239 | 0.729316 | 0.876694 |
| H | 2.666658 | -0.492349 | 2.029324 |
| H | -2.024599 | -1.646151 | 1.606861 |
| H | -3.106666 | -1.989578 | 0.251103 |
| H | -3.316095 | 0.697413 | -0.789345 |
| H | -2.650577 | -0.500520 | -1.951744 |
| K | -1.282535 | 2.142061 | 1.539261 |
| K | 1.252603 | 2.189230 | -1.411152 |
| K | 5.796066 | 0.977715 | -2.866187 |
| K | -5.877661 | 0.914682 | 2.840590 |

**Table S6** K-Glutamate

| Element | X | Y | Z |
| --- | --- | --- | --- |
| O | 1.466583 | 1.715798 | -0.878342 |
| O | -2.856333 | 0.480185 | 1.658718 |
| O | 3.054412 | 0.503186 | 0.117354 |
| O | -3.098043 | -0.794915 | -0.151225 |
| N | 1.564715 | -1.702366 | 0.129835 |
| C | -0.319974 | -0.458868 | -0.861733 |
| C | 1.192291 | -0.670790 | -0.816891 |
| C | -0.881017 | -0.086082 | 0.496596 |
| C | 1.956657 | 0.625308 | -0.490948 |
| C | -2.372494 | -0.185660 | 0.593135 |
| H | -0.810745 | -1.382845 | -1.207991 |
| H | -0.544332 | 0.325754 | -1.598825 |
| H | 1.516569 | -0.902663 | -1.856190 |
| H | -0.453519 | -0.773066 | 1.250624 |
| H | -0.573632 | 0.927981 | 0.794777 |
| H | 2.523047 | -1.510736 | 0.421815 |
| H | 1.536626 | -2.623492 | -0.298375 |
| H | -3.813261 | 0.325907 | 1.651339 |
| K | 3.331870 | 2.992024 | 0.199595 |

**Table S7** K_2_-Glutamate

| Element | X | Y | Z |
| --- | --- | --- | --- |
| O | 1.453818 | 1.670582 | -1.055962 |
| O | -2.772662 | 0.500307 | 1.771800 |
| O | 3.003169 | 0.546737 | 0.087598 |
| O | -2.986407 | -0.938631 | 0.079573 |
| N | 1.663867 | -1.739925 | 0.025154 |
| C | -0.324787 | -0.515343 | -0.788702 |
| C | 1.192065 | -0.702833 | -0.871487 |
| C | -0.796414 | -0.116992 | 0.596415 |
| C | 1.938737 | 0.610115 | -0.588329 |
| C | -2.298602 | -0.185534 | 0.822529 |
| H | -0.827794 | -1.454783 | -1.068626 |
| H | -0.622808 | 0.243025 | -1.528641 |
| H | 1.436195 | -0.913411 | -1.936935 |
| H | -0.325209 | -0.793786 | 1.331667 |
| H | -0.468699 | 0.901648 | 0.858189 |
| H | 2.616420 | -1.494025 | 0.293928 |
| H | 1.679636 | -2.644275 | -0.436994 |
| K | 2.904480 | 3.021234 | 0.442100 |
| K | -5.047875 | -0.383032 | 1.335893 |

**Table S8** K_2_-Oxalate

| Element | X | Y | Z |
| --- | --- | --- | --- |
| O | 0.743888 | -1.444624 | -0.410700 |
| O | -0.888603 | 1.175096 | -1.006414 |
| O | 1.764544 | 0.383424 | 0.381662 |
| O | -1.327075 | 0.316127 | 1.013560 |
| C | 0.749398 | -0.249059 | -0.011485 |
| C | -0.594526 | 0.480321 | 0.001795 |
| K | 3.157260 | -1.646992 | 0.125043 |
| K | -3.195806 | 1.460418 | -0.145061 |

**Table S9** K(H_2_O)_6_^+^

| Element | X | Y | Z |
| --- | --- | --- | --- |
| K | -1.247016 | 0.523733 | 0.016329 |
| O | -2.072548 | 3.083309 | -0.844502 |
| H | -2.005117 | 4.041338 | -0.767704 |
| O | -0.238561 | -2.062456 | 0.471579 |
| H | -0.649441 | -2.007095 | 1.356718 |
| O | 1.236408 | 0.010071 | 1.291137 |
| O | -3.736916 | 1.028389 | -1.245149 |
| H | -3.581443 | 1.970448 | -1.035771 |
| O | -1.016587 | -0.618070 | 2.579016 |
| H | -0.093449 | -0.295940 | 2.583111 |
| H | 1.166456 | -0.889427 | 0.915047 |
| O | -1.497668 | 1.234924 | -2.686897 |
| H | -1.241389 | 1.090172 | -3.604259 |
| H | -1.704713 | 2.838012 | -1.716215 |
| H | -2.441965 | 0.993631 | -2.609923 |
| H | -4.690989 | 0.899926 | -1.281908 |
| H | 2.174909 | 0.203459 | 1.391058 |
| H | -1.308047 | -0.655441 | 3.496552 |
| H | -0.219153 | -2.993912 | 0.226042 |
